# Gestational Diabetes in Women with Fetal Spina Bifida Repair—Influence of Perioperative Management

**DOI:** 10.3390/jcm13175029

**Published:** 2024-08-25

**Authors:** Ladina Rüegg, Ladina Vonzun, Julia Zepf, Nele Strübing, Ueli Möhrlen, Luca Mazzone, Martin Meuli, Spina Bifida Study Group, Nicole Ochsenbein-Kölble

**Affiliations:** 1 Department of Obstetrics, University Hospital Zurich, Rämistrasse 100, 8091 Zurich, Switzerland; 2 Faculty of Medicine, University of Zurich, Rämistrasse 71, 8006 Zurich, Switzerland; 3 The Zurich Center for Fetal Diagnosis and Therapy, University of Zurich, 8006 Zurich, Switzerland; 4 Department of Pediatric Surgery, University Children’s Hospital Zurich, Steinwiesstrasse 75, 8032 Zurich, Switzerland; 5 Spina Bifida Center, University Children’s Hospital Zurich, Steinwiesstrasse 75, 8032 Zurich, Switzerland; 6 Children’s Research Center, University Children’s Hospital Zurich, Steinwiesstrasse 75, 8032 Zurich, Switzerland; 7 Spina Bifida Study Group, Zurich, Switzerland

**Keywords:** fetal surgery, spina bifida, fetal myelomeningocele repair, gestational diabetes, steroids, tocolytics

## Abstract

**Background/Objectives:** Fetal spina bifida (fSB) is the most common neural tube defect, and intrauterine repair has become a valid treatment option for selected cases. If fSB repair is offered, the ideal time for surgery is from 24 to 26 gestational weeks (GWs). The preoperative steroids for lung maturation and preoperative tocolytics that are administered are known to increase the prevalence of gestational diabetes (GD), which normally occurs in about 10–15% of all pregnant women. This study assessed the prevalence, possible influencing factors, and consequences on the course of pregnancy regarding GD in this cohort. **Methods:** Between 2010 and 2022, 184 fSB cases were operated. Those patients operated on after 24 0/7 GWs received steroids before surgery. All the patients received tocolysis, and an oral glucose tolerance test was performed between 26 and 28 GWs at least 7 days after steroid administration. In 2020, we established an early postoperative mobilization protocol. The perioperative management procedures of those patients with and without GD were compared to each other, and also, the patients treated according to the early mobilization protocol were compared to the remaining cohort. **Results:** Nineteen percent were diagnosed with GD. Corticosteroids were administered in 92%. Neither the corticoid administration nor the interval between the administration and glucose tolerance test was different in patients with or without GD. Further, 99.5% received postoperative tocolytics for at least 48 h. The women with GD had significantly longer administration of tocolytics. The length of stay (LOS) was higher in those patients with GD. The gestational age (GA) at delivery was significantly lower in the cohort with GD. In the early mobilized group, we found a significantly higher GA at delivery (37.1 GWs vs. 36.2 GWs, *p* = 0.009) and shorter LOS (*p* < 0.001), and their GD rate was lower (10% vs. 20%), although not statistically significant. **Conclusions:** The GD incidence in the women after fSB repair was higher than in the usual pregnant population. Early mobilization, rapid tocolytics decrease, and shorter LOS could benefit the pregnancy course after fSB repair and may decrease the risk for GD in this already high-risk cohort without increasing the risk for preterm delivery.

## 1. Introduction

About 1 in 3000 pregnancies is affected by a fetal spina bifida aperta (fSB), which is the most common neural tube defect [1,2]. It results from the failed closure of the embryonic neural tube [3]. The occurrence of fSB is influenced by different factors such as genetic factors, folate intake, geographic location, ethnic background, maternal medication, and maternal diseases like diabetes or obesity [3,4,5,6,7,8,9]. The intrauterine repair of fSB is a valid therapeutic option for selected cases nowadays since the data of the Management of the Myelomeningocele Study (MOMS) [10] showed better outcomes of the prenatal vs. postnatal repair of fSB [10,11,12,13]. It has shown to reduce the need for ventriculoperitoneal shunt placements and to improve the long-term neurological function [10,12]. Even though fSB repair is a standard therapy in several centers, there are risks for the mother and the fetus that need to be considered [14,15].

Independently from fetal surgery and dependent on the region, gestational diabetes (GD) complicates about 11% of all pregnancies in Europe [16], with a wide range from as low as 9% in Northern Europe to up to 32% in Eastern Europe [16]. Women with GD are at higher risk for adverse pregnancy outcomes such as hypertensive pregnancy disorder, preterm delivery, small- and large-for-gestational-age (SGA and LGA) babies, and an increased cesarean section rate [17,18,19], and these complications due to gestational diabetes seem to be increasing [20]. Further, women with GD are at an increased risk for malformations of the congenital central nervous system in their offspring [21]. The risks are reported to further increase in women with pregestational diabetes compared to women with GD [22]. 

It is a known phenomenon that corticosteroids, which are mainly used during pregnancy for lung maturation in cases of threatened preterm labor, increase blood sugar levels [23]. Further, studies have shown that the prevalence of GD was higher in pregnancies where the women received tocolytics, as well as in cases where the women had decreased mobilization or were put on bedrest [24,25,26,27,28,29,30]. 

The primary goal of this study was to assess the prevalence of GD and analyze the possible influencing factors, such as the application of tocolytics and steroids, and its consequences on the course of pregnancy in this very specific cohort. Pregnancies with and without GD after fSB repair were compared. Secondarily, the cohort of fSB repair was also compared to patients who were treated with tocolytics and steroids for threatened preterm labor without fetal surgery at our center. 

## 2. Materials and Methods

### 2.1. Patients and Study Design

This is a retrospective single-center cohort study. Between December 2010 and April 2022, 184 women had open fSB repair at the Zurich Center for Fetal Diagnosis and Therapy (Home | SwissFetus accessed on 16 July 2024). Two women withdrew consent, leaving data of 182 cases for analysis.

The criteria for eligibility for the surgery, peri- and postoperative management, as well as the surgical techniques have been published in detail previously [11,12]. All women were treated according to our protocol: Patients were hospitalized three days prior to surgery. When surgery was planned at a gestational age (GA) greater than 24 weeks of gestation (GWs), they received lung maturation with betamethasone 12 mg on days one and two of hospitalization. Surgery then took place on day three, when lung maturation was considered effective. Magnesium infusion for fetal neuroprotection was administered perioperatively. One hour before surgery, tocolysis with tractocile was started, initially with a bolus of 6.75 mg and continued with 75 mg in 90 mL at a running rate of 24 mL/h for the first 3 h and then continuously with 8 mL/h for at least 48 h postoperatively. After surgery, women were monitored in an intermediate care unit for 2 days where contractions were continuously monitored by tocography (IntelliSpace, Perinatal Information system, Philips AG Healthcare, Horgen Switzerland). If periodic contractions were registered, additional tocolysis with hexoprenaline was administered, usually starting with a dose of 100 mcg in 500 mL and an application rate of 30 mL/h (dose was increased if needed). Postoperatively, registration of contractions mainly relied on tocography as women still had epidural anesthesia and thus, in most cases, did not feel contractions. 

After transferring patients to the prenatal ward, cardiotocography was performed twice daily to monitor the fetal heart rate and contractions. Additionally, ultrasound was performed once a week. Tocolytics were reduced as soon as the patients were stable and the epidural was stopped. When the intravenous tocolytics were removed, an oral tocolysis with nifedipine was started and administered until the scheduled primary cesarean delivery at 37 GWs [31]. 

The first 153 cases had a strict “no mobilization” protocol after surgery: women were on bedrest for the first 24 h postoperatively. After 24 h, they were instructed to sit up on the edge of the bed. Only after 48 h they were instructed to stand up for the first time. Since December 2020, from patient 154 onwards, we started with an adapted early mobilization protocol. This allowed women to sit within 6 h, if possible, or at the latest on the first postoperative day. In case of adequate pain control and absent complications, they were instructed to stand up within the first postoperative day. Additionally, women received instructions regarding early mobilization from physiotherapists. 

If the oral glucose tolerance test was not performed prior to surgery, it was performed postoperatively with a minimum time lapse of 7 days following lung maturation. 

At the very beginning of performing fSB repair at our center, women were hospitalized for 3–4 weeks after fetal surgery. Nowadays, if clinically stable, women are discharged 10–14 days after fSB repair with planned re-hospitalization around 34 GWs and scheduled cesarean delivery at 37 GWs. 

Data were reviewed for baseline characteristics, administration of lung maturation and tocolytics, duration of tocolytics, time between lung maturation and oral glucose tolerance test, length of stay (LOS), and adverse pregnancy outcomes (gestational hypertension, preeclampsia, intrauterine growth restriction (IUGR), marcosomia, and preterm delivery). Additionally, neonatal outcomes were analyzed, particularly gestational age (GA) at delivery, birth weight, pH, and Apgar score. 

Primarily, we studied the incidence of GD in the fSB repair cohort and compared women with and without GD. We then analyzed whether increasing experience with reducing the postoperative hospitalization time and adaptation of the perioperative management and postoperative protocol had an influence on the occurrence of GD in this cohort. To evaluate whether those factors impacted GD rate, we compared three groups: group 1 (no early mobilization protocol) operated on between 2010 and 2017, group 2 (no early mobilization protocol) operated on between 2017 and 2020, and group 3 (early mobilization protocol) operated on between 2020 and 2022. 

Secondarily, the fSB repair cohort was compared to a “preterm cohort”, a group of women who were hospitalized at the same center for threatening preterm labor and who received steroids for lung maturation and/or tocolytics. Their data regarding the rate of GD and the influence of steroids and tocolytics were compared to those of women after fSB repair. 

### 2.2. Data Analysis

Descriptive statistics were performed with SPSS version 25.0 (IBM, SPSS Inc., Chicago, IL, USA). Quantitative data are presented as median (IQR) or mean +/− standard deviation (SD) depending on data distribution, analyzed using Shapiro–Wilk Test. Categorical data were compared by chi-squared test and provided as percentages. Continuous data were compared using a T-Test or Mann–Whitney U Test depending on data distribution. Statistical significance was provided with *p* < 0.05. 

The study was conducted in accordance with the principles of the Declaration of Helsinki and the International Conference on Harmonization E6 (Good Clinical Practice) guidelines. All included women provided their written informed consent. The study was conducted after the approval of the local ethics commission (KEK-ZH Nr. 2021-01101). 

## 3. Results

### 3.1. Primary Outcomes

The baseline characteristics of the fSB repair cohort and the preterm cohort are listed in Table 1. In the fSB repair cohort, corticosteroids were administrated in 92% of all the women. Further, 99.5% received postoperative tocolytics for at least 48 h. The most frequently used tocolytics were tractocile (90%), hexoprenaline (73%), and nifedipine (95%).

In the fSB repair cohort, 19% of the women were diagnosed with GD. The median LOS was 18 days (15–31 days). The data of the women with and without GD after fSB repair are listed in Table 2. The interval between the administration of corticosteroids and the glucose tolerance test was similar in those cases with and without GD (*p* = 0.18). The women with GD had significantly longer tocolysis with tractocile (*p* = 0.01) and hexoprenaline (*p* = 0.002). The LOSs were significantly longer in the patients with GD (*p* = 0.001). The GA at delivery was significantly lower, with 35.6 GWs (34.1–36.7 GWs) vs. 36.3 GWs (35.0–37.4 GWs) in the cohort with GD (*p* = 0.01). The neonatal outcomes and pregnancy complications did not differ between the two groups (Table 2). 

### 3.2. Changes in the Perioperative Protocol over the Years 

Although not statistically significant, the prevalence of GD steadily decreased from 23% in those cases operated on between 2010 and 2017 (group 1) to 19% in the cases operated on between 2018 and 2020 (group 2) and to 10% in the cases operated on between 2020 and 2022 (group 3), as shown in Table 3. 

There was no change in the GA at operation between the groups. Significantly fewer women received steroids in the first group compared to the other two groups due to a few cases being operated on before 24 GWs. The duration of atosiban and hexoprenaline administration was significantly longer in group 1 compared to group 3 (*p* < 0.001 and *p* = 0.007, respectively). Over the years, the postoperative LOS decreased significantly from group 1 to group 3 (*p* < 0.001), whereas the GA at delivery increased from group 1 to group 3 (*p* = 0.02). Also, with implementing the early mobilization protocol, the LOS was halved (29 ± 20 days vs. 14 ± 6 days, *p* < 0.001), while the GA at delivery increased, as shown in Table 4.

### 3.3. Secondary Outcomes

Between 2010 and 2022, 51,736 children were born at the University Hospital Zurich. Further, 7039 women (14%) were diagnosed with GD. In the preterm cohort, consisting of 4755 women who were hospitalized due to threatening preterm labor, 2486 (52%) received steroids for lung maturation, 1011 (21%) received hexoprenaline, and 256 (5%) received tractocile for tocolysis. Moreover, 19% of the women in this preterm cohort were diagnosed with GD, which was comparable to the cohort of women with fSB repair (*p* = 0.86). 

## 4. Discussion

This study shows that the prevalence of GD in women undergoing fSB repair is higher in comparison to the general pregnant population but comparable to women hospitalized for threatening preterm labor. 

The women diagnosed with GD in the fSB cohort had significantly longer tocolysis as well as LOSs compared to the women without GD in the fSB cohort. The early mobilization protocol had a positive effect on the GD rates without affecting the GA at delivery. 

The adverse effects of corticosteroids and tocolytics, especially beta-adrenergic agents, on the development of GD were described early on [25,26]. However, in our fSB cohort, the results did not show an influence of corticosteroid application on the prevalence of GD. A study from Hong et al. showed that hyperglycemia after the injection of corticosteroids was only short-lasting and was mainly elevated on the first day of administration [33]. This supports our findings that, after the mentioned interval of 7 days between the administration of steroids and the glucose tolerance test, the influence of steroids on GD diagnosis is negligible. 

However, we noticed a significantly longer use of tocolysis, especially applying hexoprenaline, in those women with versus without GD. 

A systematic review by Malaza et al. [22] looked at several pregnancy complications in women with diabetes, including pregestational diabetes and GD. The studies reported an increased risk for preterm birth in women with diabetes [34]. This risk is the highest in women with pregestational diabetes [22], but the risk for preterm birth is also increased in women with GD. 

A study by Stogianni et al. also supports this finding. The risk for preterm delivery was significantly higher in those women with diabetes than without (21% vs. 6%) and also in the women with pregestational diabetes compared to the women with GD (38% vs. 12%), with the highest risk being in the women with type 2 diabetes, followed by type 1 diabetes and GD [35]. 

Not only diabetes but also fetal surgery increases the risk for preterm birth [36]; this is why the women in this study represent a high-risk cohort.

A randomized controlled trial by Barakat et al. [37] showed a decreased risk of GD in women who exercised regularly during pregnancy. A meta-analysis by Sanabria-Martinez et al. [38] supports those findings since they equally showed that physical exercise, especially throughout a pregnancy, was associated with a decreased risk of GD [38]. This might also explain the finding in our fSB cohort, where the women with longer LOSs, and therefore decreased physical activity, had higher rates of GD. This was also observed in the control cohort of women hospitalized for threatening preterm delivery. Bedrest for high-risk pregnancies is widely performed, even though multiple studies, such as a Cochrane analysis from Sosa et al. [39], did not show a clear benefit from bedrest. Also, the Society for Maternal-Fetal Medicine (SMFM) recommends against the routine activity restrictions in women at risk of preterm birth [40].

Further, our group 1 also underwent longer administration of tocolysis. This shows that this topic is complex and GD in this cohort is influenced by several factors that need to be considered in the treatment regimen. Our data show that prudent but early mobilization in the high-risk cohort of fSB repair can improve the pregnancy outcome without increasing the risk for preterm delivery. This is in accordance with a review of Palacio et al. [41], which looked at bedrest exercise that may reduce the physiological deconditioning in hospitalized individuals with high-risk pregnancies, and showed that even lower-intensity physical activity is beneficial. Instead of prohibiting the overall activity in such a high-risk cohort, modified activity seems to be important [41,42]. 

We need to be aware of the high risk for preterm birth in this special cohort, with GD possibly even increasing the risk for preterm delivery. However, when comparing the results according to the different time points and postoperative protocols (groups 1–3), the results showed that, in cases of early mobilization (group 3), the LOS was decreased without risking preterm delivery. This study’s results showed that the LOS could be decreased while even reaching a higher GA at delivery and possibly being beneficial on the development of GD as well. 

### Strengths and Weaknesses

This is, to our knowledge, the first study that systematically addresses a common obstetric complication, namely GD, in a high-risk cohort of women undergoing fetal surgery. 

Negatively, the number of women in the early mobilization protocol was still small. To strengthen these results, the findings need to be reanalyzed once further women are included in the early mobilization protocol group. Also, the retrospective study design represents another limitation. 

## 5. Conclusions

The incidence of GD in women after fSB repair was higher than in the average pregnant population but equal to women hospitalized for preterm labor. 

Although GD in this cohort is probably multifactorially conditioned, factors such as early mobilization and/or a reduction in the tocolysis duration with the consequent shortening of the LOS may reduce the GD incidence in women after fSB repair without risking preterm delivery, a common complication after fetal surgery. Thus, stringent early mobilization and timely tocolysis reduction are recommended to positively influence the pregnancy course after fSB repair. 

## Figures and Tables

**Table 1:** Baseline characteristics of the fSB repair cohort in comparison with the preterm cohort.

Baseline Characteristics	N = 182 fSB Repair Cohort	N = 4755 Preterm Cohort	*p*-Value
Maternal age, [years]	32.5 ± 4.8		
BMI, [kg/m^2^]	27.0 ± 5.3	23 ± 4.7	<0.001
GA at surgery, [GWs, median (IQR)]	25.3 (24.1–25.5)	-	-
Administration of corticosteroids, N (%)	168 (92)	2486 (52)	<0.001
Administration of tocolytics, N (%) and duration [days, median (IQR) or mean ± SD] Tractocile hexoprenaline	163 (90), 6 (4–10) 132 (73), 19 ± 19	256 (5) 1011 (21)	<0.001 <0.001
Duration of hospitalization, [days, median (IQR)]	18 (15–31)	6 (4–18)	<0.001
GA at delivery, [GWs, median (IQR)]	36.0 (34.0–37.0)	36.4 (33.1–38.3)	0.49

BMI = body mass index; GA = gestational age; GWs = gestational weeks; IQR = interquartile range; SD = standard deviation.

**Table 2:** Comparison of patients with and without GD in the fSB repair cohort.

	GD (N = 34)	No GD (N = 148)	*p*-Value
BMI at surgery, [kg/m^2^]	28 ± 7	27 ± 5	0.52
Corticosteroids for lung maturation, [N] (%)	32 (94)	136 (92)	1.0
GA at at lung maturation, [GWs, mean ± SD]	24.9 ± 0.9	24.8 ± 0.8	0.53
GA at surgery, [GWs, median (IQR)]	25.3 (24.2–25.5)	25.3 (24.1– 25.5)	0.8
Total duration of tocolytics Tractocile, [days, median (IQR)] Hexoprenalin, [days, mean ± SD]	8 (5–13) 29 ± 22	6 (4–9) 16 ± 17	0.01 0.002
GA at glucose tolerance test, [GWs, median (IQR)]	27.1 (26.4–27.9)	26.7 (26.0–27.4)	0.04
Interval corticosteroid application to glucose tolerance test, [days, median (IQR)]	16 (13–19)	15 (12–18)	0.18
Postoperative LOS, [days, median (IQR)]	29 (17–55)	17 (15–28)	<0.001
GA at delivery, [GWs, median (IQR)]	35.4 (34.1–36.7)	36.3 (35.0–37.4)	0.01
Apgar score, [5 min, median (IQR)]	9 (8–9)	9 (8–9)	0.57
Birth weight, [32]	2556 ± 451	2563 ± 558	0.94
Percentile birthweight, [N] (%) <3. 3–9. 10–24. 25–74. 75–89. 90–97. >97.	1 (3) 4 (12) 5 (15) 9 (26) 10 (29) 4 (12) 1 (3)	2 (1) 8 (5) 35 (24) 49 (33) 45 (30) 5 (3) 1 (1)	0.2
Pregnancy complications, [N] (%) Hypertension Preeclampsia	0 (0) 1 (3)	1 (1) 4 (3)	0.8

BMI = body mass index; GA = gestational age; GWs = gestational weeks; LOS = length of stay; IQR = interquartile range; SD = standard deviation.

**Table 3:** Comparison of groups 1, 2, and 3 according to the year of fSB repair and early mobilization protocol.

	1 2010–2017 N = 70	2 2018–2020 N = 81	3 2020–2022 N = 31 (early Mobilization)	*p*-Value
Gestational diabetes [N] (%)	16 (23)	15 (19)	3 (10)	0.29
GA at surgery [GWs, median (IQR)]	24.9 (24.4 -25.2)	25 (24.4 -25.4)	25.3 (24.1–25.5)	0.41
Corticosteroid application [N] (%)	57 (81)	80 (99)	31 (100)	<0.001
Duration of tocolytics Tractocile [days, median (IQR)] Hexoprenalin [days, mean ± SD]	7 (4–11) 20 ± 18	6 (4–10) 21 ± 18	5 (4–9) 11 ± 9	<0.001 0.007
LOS [days, median (IQR)]	33.3 ± 22	25 ± 19	14 ± 6	<0.001
GA at delivery [GWs, median (IQR)]	36.2 (34.3–37.0)	36.3 (33.0–37.6)	37.1 (35.0–37.1)	0.02

GA = gestational age; GWs = gestational weeks; LOS = length of stay; IQR = interquartile range; SD = standard deviation.

**Table 4:** Differences after initiating a strict early mobilization protocol.

	Early Mobilization (N = 31)	Bedrest for First 24 h (N = 151)	*p*-Value
Gestational diabetes, [N] (%)	3 (9.7)	28 (18.5)	0.16
GA at surgery, [GWs, median (IQR)]	25.3 (24.1–25.5)	25.2 (24.1–25.4)	0.30
Corticosteroid application, [N] (%)	31 (100)	137 (90.7)	0.13
Duration of tocolytics Tractocile [days, median (IQR)] Hexoprenalin [days, mean ± SD]	5 (4–9) 11 ± 9	6 (4–10) 21 ± 20	0.54 0.03
LOS, [days, median (IQR)]	14 ± 6	29 ± 20	<0.001
GA at delivery, [GWs, median (IQR)]	37.1 (35.0–37.1)	36.2 (34.0–37.1)	0.03
birth weight, [gram, mean ± SD]	2586 ± 598	2557 ± 529	0.79

GA = gestational age; GWs = gestational weeks; LOS = length of stay; IQR = interquartile range; SD = standard deviation.

## Data Availability

The data presented in this study are available on request from the corresponding author. The data are not publicly available due to privacy reasons.
